# System-wide detection of protein-small molecule complexes suggests extensive metabolite regulation in plants

**DOI:** 10.1038/srep42387

**Published:** 2017-02-13

**Authors:** Daniel Veyel, Sylwia Kierszniowska, Monika Kosmacz, Ewelina Maria Sokolowska, Aenne Michaelis, Marcin Luzarowski, Jagoda Szlachetko, Lothar Willmitzer, Aleksandra Skirycz

**Affiliations:** 1 Max Planck Institute of Molecular Plant Physiology, Potsdam, Germany

## Abstract

Protein small molecule interactions are at the core of cell regulation controlling metabolism and development. We reasoned that due to the lack of system wide approaches only a minority of those regulatory molecules are known. In order to see whether or not this assumption is true we developed an effective approach for the identification of small molecules having potential regulatory role that obviates the need of protein or small molecule baits. At the core of this approach is a simple biochemical co-fractionation taking advantage of size differences between proteins and small molecules. Metabolomics based analysis of small molecules co-fractionating with proteins identified a multitude of small molecules in Arabidopsis suggesting the existence of numerous, small molecules/metabolites bound to proteins representing potential regulatory molecules. The approach presented here uses Arabidopsis cell cultures, but is generic and hence applicable to all biological systems.

Recent years brought significant advances in so called omics techniques allowing for simultaneous quantification of thousands of biological molecules including transcripts, proteins, and metabolites^1^. These molecules present building blocks of life but it is their interactions that enable life. Large-scale analysis of molecular complexes is thus one of the next great challenges to be addressed. Among these, the analysis of protein – metabolite interactome (PMI), irrespective of its high potential importance for both basic research (i.e. identifying novel signaling molecules^2^) and translational research (i.e. identifying lead compounds for drugs^3^), received comparatively little attention. The reason for this is twofold: First, the monitoring and identification of metabolites is particularly troublesome considering the large diversity of small molecules. Second, no generally applicable approaches allowing the system wide monitoring of protein – metabolite interactions have been described. Most existing approaches can be divided into those using metabolites as baits to “fish out” interacting proteins or *vice versa*. Immobilizing small molecules to identify protein receptors is a straight forward approach and is being used since decades^4^,^5^. Cross linking approaches represent another way of pulling protein receptors of modified small molecules^6^. Conversely, several studies discovered protein binding small molecules by tagging proteins of interest^2^,^3^,^7^. The downside of these methods is the modification of the prey which may influence the interaction. Methods avoiding immobilization of either interaction partner exist, but are so far restricted to samples of highly reduced complexity comprising predefined proteins or small molecules^8^,^9^,^10^.

Here we show an alternative approach that relies on global analysis of protein bound small molecules using metabolomics technologies. The basic principle of our approach is based on the supposition that small molecules/metabolites interacting with proteins and forming stable complexes will fractionate together. Thus when applying any type of size separation (size exclusion chromatography, size filtration and the like) both should appear in the high molecular weight fraction. In contrast, non-bound metabolites will remain in the low molecular weight fraction.

## Results

**Figure 1:**
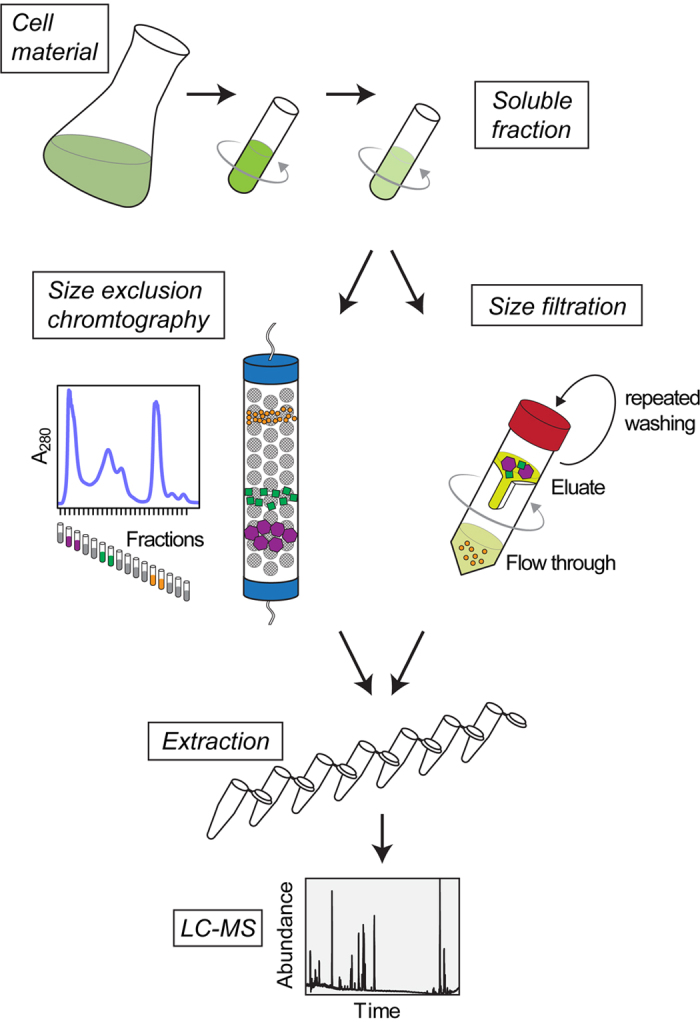
Experimental workflow. Cells were extracted using native buffer. Native soluble fraction was obtained by ultracentrifugation step. Size filtration was performed using 10 kDa spin columns. Alternatively, protein-metabolite complexes were separated from free metabolites using SEC. Collected samples were dried and subjected to MTBE-Methanol-Water extraction. Polar metabolites were quantified by LC/MS.

**Figure 2:**
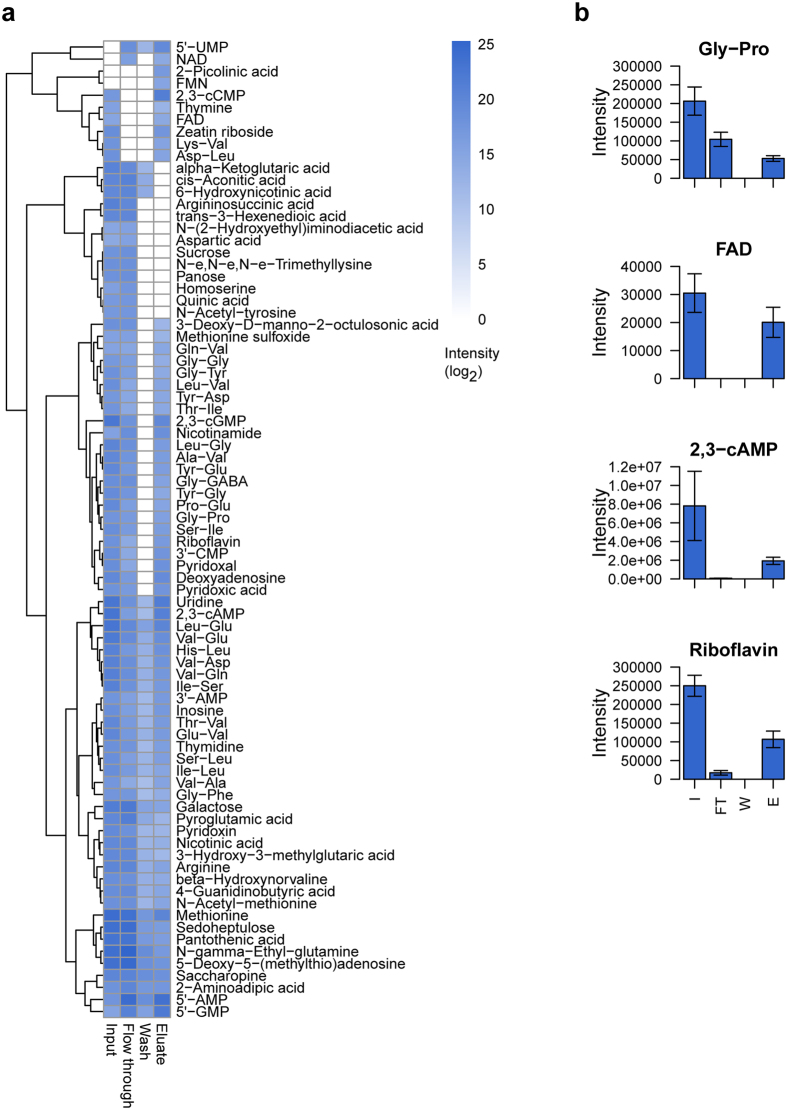
Size filtration separates protein bound from free small molecules. (**a**) Heatmap presentation of 81 small molecules identified in the size filtration experiments in input, flow through, wash, and eluate samples. Aliquots of the different fractions were collected, boiled to denature proteins, dried and re-extracted using MTBE-Methanol-Water (see Methods). Input represents starting material, metabolites found in the flow through and wash are referred to as free, whilst those measured in the eluate as protein bound (mean, n = 5). Note that the absence of a metabolite in the input, as e.g. FMN, may be caused by ion suppression common for the complex samples on LC/MS. (**b**) Exemplary bar plots of individual metabolites showing their intensity distribution in the different fractions (mean and standard deviation, n = 5).

To see whether or not this assumption holds true, and to obtain a first insight into the wealth of protein bound metabolites, we applied our approach to plant cell suspension culture extracts. To this end, *Arabidopsis thaliana* cell cultures^11^ were lysed by physical stress and subjected to an ultracentrifugation step (cf. Methods). The supernatant containing essentially soluble proteins and metabolites (later referred to as input) was subsequently subjected to a size filtration spin column with a 10 kDa cutoff to separate proteins (high molecular weight fraction) from free metabolites (low molecular weight fraction, flow through) (Fig. 1). Subsequently the fraction retained on the filter (mostly proteins) was washed thoroughly in order to remove any non-bound metabolites (wash). To release metabolites which were non-covalently bound to proteins heat denaturation was applied (elution). All samples (input, flow through, wash and eluate) were subsequently dried and re-extracted using Methyl-tert-butyl ether (MTBE)-Methanol-Water, which separates proteins (in the pellet) from polar and lipid small molecule fractions^12^. For the purpose of this work, polar compounds were subsequently analyzed by LC/MS (Fig. 1). The above described filtration procedure relies on two assumptions: (1) that the protein metabolite complex is stable enough to be retained on a spin column whereas all non-bound metabolites will be in the flow-through, and (2) that upon heat denaturation metabolites not covalently bound to proteins will be released. Overall, LC/MS analysis of input, flow through, wash and eluate samples resulted in 81 metabolic features which could be annotated to a metabolite using our in-house reference compounds library (Supplementary Table S1). As expected, the flow through contained many (unbound) metabolites and the metabolite content decreased in the washing. Strikingly, after heat treatment many metabolites, while being absent in wash samples, were detectable in the eluate. This strongly suggests that this large fraction of metabolites is indeed forming stable complexes with proteins (Fig. 2). Among these were well-known ligands such as cyclic nucleotides (cGMP, cAMP, cCMP), co-factors (FAD, NAD, FMN), and dipeptides (Fig. 2, Supplementary Table S1). These results indicate that in the biological system analyzed numerous metabolites/small molecules form a non-covalent but stable complex with proteins.

To challenge our filtration procedure we addressed two major points that in our opinion could raise concern. First, heat denaturation may lead to both false positives and negatives by influencing small molecule chemical composition e.g. by promoting hydrolysis. To test this, we compared results of the elution fraction with or without heat denaturation prior to MTBE-Methanol-Water extraction. Comparison of the metabolites obtained by the two methods did not reveal major differences showing a high correlation (R = 0.99) (Supplementary Fig. S1), suggesting that both approaches could be used interchangeably. Second, salt used in the lysis and washing buffers can affect interactions. Too low salt concentrations may promote unspecific binding, while too high concentrations will destroy interactions by interfering with ionic bonds and by promoting protein denaturation. To find optimal conditions we compared elution profiles obtained using three different NaCl concentrations: 0.05 M, 0.5 M and 1.5 M. As expected high salt conditions (1.5 M) were disruptive for most interactions analyzed and fewer metabolites could be found in the eluate (Supplementary Fig. S2a). Shifting from 0.05 M to 0.5 M NaCl had a minor effect on the number of identified metabolites, but still decreased their quantity (Supplementary Fig. S2b). Thus for consecutive experiments we used intermediate 0.15 M concentration (see SEC experiments).

**Figure 3:**
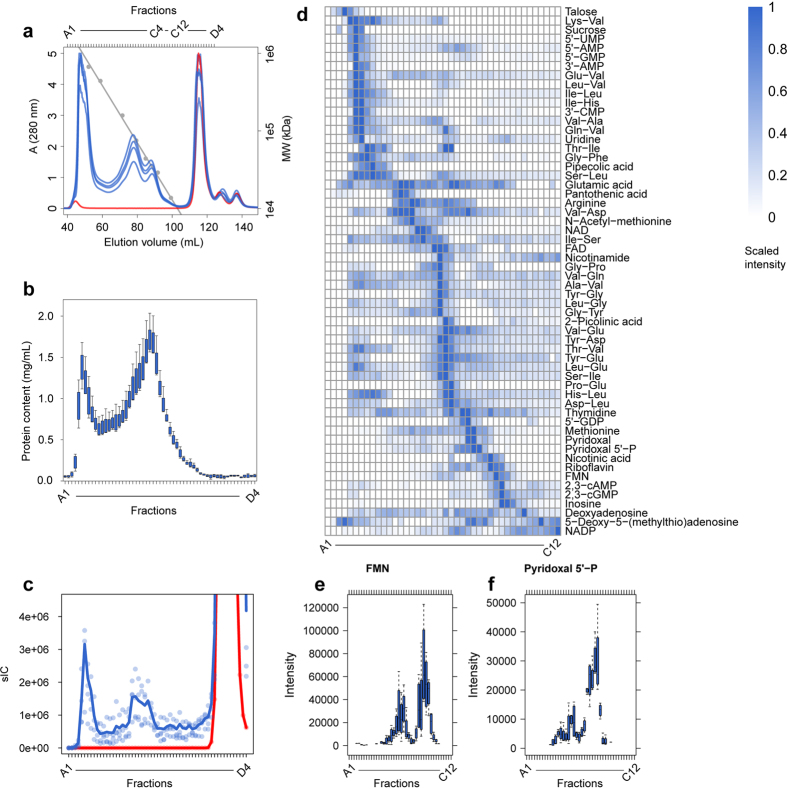
SEC separates protein bound from free small molecules. (**a**) Chromatograms of the absorption at 280 nm of four SEC replicates (blue) and the non-protein control (red). The approximate molecular weight distribution as determined from a standard curve is plotted in grey. (**b**) Protein content of the 57 analyzed fractions from SEC analysis. (**c**) Summed ion count across SEC fractions identified small molecules plotted in (**d**) of independent experiments (circles) and their mean (lines). (**d**) Heat map of SEC profiles of 57 identified small molecules in fractions greater 10 kDa (scaled intensity of the mean of n = 4 experiments). Exemplary SEC profiles of the co-factors FMN (**e**) and Pyridoxal 5′-phosphate (**f**). Abbreviations: sIC: summed ion count, A: absorption, MW: molecular weight.

In a next step we decided to move to an alternative size fractionation approach. To this end we applied size exclusion chromatography (SEC) to the native protein-metabolite extract using a column which separates molecular complexes from approximately 600 kDa to 10 kDa^13^,^14^ (Fig. 1). The chromatogram of the absorption at 280 nm indicates reproducible separation of complexes present in our input sample by SEC (Fig. 3a). As expected, the protein content in the collected fractions showed separation of protein containing molecular complexes, whereas no protein was detectable in fractions later than C12 corresponding to a molecular weight smaller than 10 kDa (Fig. 3a,b, Supplementary Fig. S1). To see whether or not the protein fractions contained metabolites/small molecules which based on our first principles, could only be understood if assuming stable protein-metabolite complexes, we analyzed 57 fractions for occurrence of polar metabolites by LC/MS. According to the results obtained from size filtration we detected the majority of metabolic features as non-protein bound and therefore eluting after one total mobile phase volume of the column (Fig. 3c). In addition, and more important however we detected specific elution profiles of small molecules in fractions representing theoretical MWs between 10 to 600 kDa.

Three features of small molecule elution profiles are worth commenting. Firstly, the SEC results confirmed to a large extent the results of the size filtration experiment described above. Indeed 51 of the annotated metabolites identified as binding to proteins from the size filtration experiment were also observed in the SEC data distributed along the whole separation range (Fig. 3d). Secondly, for most metabolites/small molecules we observed their occurrence in distinct fractions and not throughout all SEC fractions which is a clear indication of a specific binding to one or more proteins eluting in these fractions. Thirdly and most important for proof of concept, we observed metabolites well known to interact with proteins most notably co-factors such as FMN or NAD to display distinct peaks in the separation range indicating the presence of multiple though specific binding to protein(s) (Fig. 3e,f).

To assure that differential eluting metabolites are truly metabolite-protein complex derived we performed a control experiment with a protein-free sample. To this end, we precipitated proteins from the input sample with 80% acetone and reconstituted the small molecules in lysis buffer before applying it to the SEC column. In line with SEC separating molecular complexes based on their size we did not observe any significant differential metabolic features across the SEC fractions (Fig. 3c) but all metabolites appeared after one total mobile phase volume as expected for free small molecules.

Thus the data presented up to here show elution of a small proportion of metabolites present in a cell free extract in the high-molecular weight fraction which is unexpected whereas the by far largest part of metabolites elute in the low-molecular weight area which is expected. Furthermore the elution of metabolites in the high-molecular weight fraction is dependent on the presence of proteins. These observations together with the filtration experiments described above suggest that the metabolites appear in the high-molecular weight fraction because they are bound to proteins.

**Figure 4:**
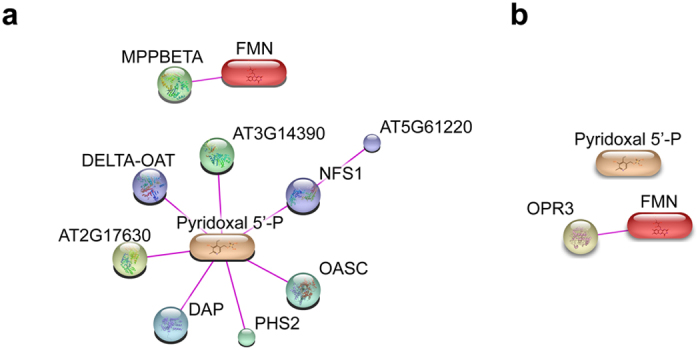
SEC approach can retrieve known protein – ligand complexes. Network representation of information on experimentally confirmed FMN and Pyridoxal 5′-phosphate binders as an output of the STITCH 5 database. The STITCH 5 database was queried against Pyridoxal 5′-phosphate and FMN together with the respective specific proteins detected in SEC fractions (**a**) B05 (203 proteins) and (**b**) C01 (133 proteins). Specificity of proteins was determined by presence in proteomics data in both replicates of one, but absence in the other fraction. Only experimentally confirmed interactions of the queried metabolites and proteins above score 0.7 were kept. For the individual scores and gene annotations of the shown proteins see Supplementary Table S6.

If this is true and if this approach is going to be useful to monitor system-wide protein metabolite complexes one would request that known metabolite-protein interactions should at least partially be covered. To this end we performed two types of experiments: In a first experiment we tested all protein fractions via an antibody for the presence of a given protein. Glutamine Synthetase (GLN) is well-known to bind glutamate. To see if GLN is migrating together with one of the glutamate peaks, we probed the fractions of SEC with an antibody against GLN in a Western blot experiment. As evident from Supplementary Figure S4, the major peak of GLN coelutes with one of the peaks of glutamate. This major GLN peak appeared from the SEC column at approximately 390 kDa and hence likely represents the homodecamer of GLN^15^. Several other proteins which in addition were recognized by the antibody may represent another GLN isoform or cross-reacting proteins (Supplementary Figure S4). Pyridoxal 5′-phosphate and Flavin mononucleotide (FMN) represent two well-known co-factors. Their elution profiles in the SEC experiment display the highest intensity in the high molecular weight area in fractions B05 and C1 respectively (Fig. 3e,f). In a second experiment we thus performed a proteomics analysis of these two fractions to see whether or not we can detect proteins which are known to bind either of the two co-factors (Supplementary Table S5). Within the proteins detected in fraction B05 (a total of 203 proteins) 8 proteins were detected which are described to interact with Pyridoxal 5′-phosphate (Fig. 4 for the network of these proteins as based on the STITCH^16^ database) whereas in fraction C1 (where we detected a total of 133 proteins) we did not detect any protein interacting with Pyridoxal 5-phosphate but did detect one protein known to interact with FMN (Fig. 4) again based on the STITCH database. Thus taken together these experiments demonstrate that metabolites respectively co-factors coelute with proteins known to interact with these proteins thus strongly lending further support to the validity of our approach.

In summary, we provide evidence that biological systems (here: *Arabidopsis thaliana* suspension cultures) contain a multitude and up to now unexplored number of small molecules/metabolites which form stable complexes with proteins and thus represent candidates of small molecules displaying a signaling/regulatory function. The approach described here will largely improve the identification of novel metabolite-protein interactions by its (1) dispensability of protein and metabolite baits (2) proteome/metabolome wide scale (3) relative simplicity and (4) generic nature making it suitable across biological systems. Nevertheless, our method is not free of limitations. As with other techniques starting with a native lysate there is always a chance of false positives related to mixing proteins and metabolites present in separate organelles and/or used buffering conditions. Obtained binding may be therefore specific in our experiment but not necessarily biologically relevant. Moreover, co-elution data obtained from SEC provide indication of binding event but require independent validation.

## Methods

### Growth of Arabidopsis cell cultures

Arabidopsis cells cultures^11^ were grown in MSMO medium (Sigma Aldrich) supplemented with 3% sucrose, 0.05 mg/L kinetin and 0.5 mg/L 1-naphthaleneacetic acid on orbital shaker at 130 rpm in the light. Cells were passaged weekly to fresh medium and harvested during logarithmic growth using rapid filtration and liquid nitrogen snap freezing.

### Cell lysis and preparation of soluble protein fraction

Frozen cells were grinded with mortar and pestle or a Retsch mill (Retsch GmbH, Haan, Germany) for 4 times 1 min at 30 rps. 1.5 mL (for size filtration) or 0.7 mL (for size exclusion chromatography) of lysis buffer (50 mM Tris-HCl pH 7.5, 500 mM NaCl, 1.5 mM MgCl_2_, 5 mM DTT, 1 mM PMSF, 1x Protease Inhibitor Cocktail (Sigma-Aldrich), 0.1 mM Na_3_VO_4_ and 1 mM NaF) were added per 1 g of cells. In SEC experiments, 50 mM ammonium bicarbonate-HCl pH7.5 was used instead of Tris-HCl as buffering agent and the NaCl content was lowered to 150 mM. After thawing on ice the extract was filtered through miracloth and subsequently centrifuged 10 min at 3452 g, 4 °C. Ultracentrifugation 45 min at 35000 rpm (max 165052 g, avg 125812 g), 4 °C was used to prepare the soluble fraction.

### Size filtration

2.5–3 mL of soluble fraction (see above) were filtered using Amicon 10 kDa Ultra centrifugal filter units (Millipore). At this stage 400 μL aliquots of input and flow through were kept for metabolic analysis. Two washing steps, first using 5 mL and second 1.5 mL of wash buffer (50 mM TrisHCl pH 7.5, 500 mM NaCl, 1 196.5 mM MgCl_2_) were applied to get rid of the remaining free metabolites. Approximately 1.5 mL of wash buffer was added to the column to cover the filter and a 10 min, 100 °C treatment was used to denature proteins and to dissociate protein-metabolite complexes. 1 mL–1.2 mL aliquots from second wash step and eluate were dried and kept for metabolic analysis. Centrifugation steps were performed at 3452 g for 15–30 minutes.

### Size exclusion chromatography

2.5 mL of soluble fraction corresponding to 50 mg of protein were used for the separations. SEC was performed with a HiLoad 16/600 Superdex 200 prep grade column (GE Healthcare Life Science, Little Chalfont, UK) connected to an ÄKTA explorer 10 (GE Healthcare Life Science, Little Chalfont, UK) operating at 4 °C. The flow rate was set to 0.8 mL/min. 57 fractions of 1.5 mL were collected from 40 to 125.5 mL elution volume of which 1 mL was dried in a speed-vac overnight and stored at −80 °C for metabolomic analysis. For the protein free control experiment, 50 mg of protein of the soluble fraction was precipitated with 80% acetone at −20 °C for 5 h. After pelleting denatured proteins by centrifugation at 3452 g for 20 min at 4 °C, the supernatant was dried overnight in a speed-vac. Small molecules were resuspended the next day in the original volume of lysis buffer and used for SEC.

### Metabolite extraction and LC/MS metabolomics

Samples were extracted as described by^12^. This method uses a Methyl tert-butyl ether (MTBE)/Methanol/Water solvent system to separate proteins, lipids, and polar compounds into pellet, organic, and aqueous phase, respectively. After extraction, the aqueous phase was dried in a speed-vac and stored at −80 °C until L C/MS analysis. Samples were measured using ultra-performance liquid chromatography coupled to an Exactive mass spectrometer (Thermo-Fisher; http://www.thermofisher.com) in positive and negative ionization mode as described in ref. 12. Processing of chromatograms, peak detection, and integration were performed using REFINER MS 9.0.4 (GeneData; http://www.genedata.com). Processing of mass spectrometry data included retention time alignment, peak detection, removal of the isotopic peaks, as well as chemical noise. Obtained metabolic features (m/z at a given retention time) were queried against an in-house reference compound database allowing 0.15 min retention time and 3 ppm m/z deviation. For metabolites that showed a higher ppm error in standard compound measurements we allowed a corresponding deviation of up to 10 ppm (as indicated in the Supplemental Tables).

### Data analysis and plotting

LC/MS peak lists obtained as Refiner MS output were subsequently analyzed using EXCEL (Microsoft Corp.) or R (http://www.r-project.org/). Heatmaps were produced using the heatmap package in R with default settings on appropriately transformed data. All other plots were done with the basic plotting function in R. Figures were post processed with Adobe Illustrator (Adobe Systems) when needed.

### LC-MS proteomics analysis

Pellets recovered after metabolite extractions were used for mass spectrometric protein analysis. Proteins were dissolved in 100 μL of 6 M urea/2 M thiourea in 40 mM ammonium bicarbonate buffer. An equivalent to 100 μg protein diluted to 46 μL was treated with 5 mM dithiothreitol for 30 min at RT, followed by cysteine alkylation with 15 mM iodacetamide for 20 min at RT in the dark. Proteins were digested using a LysC/Trypsin Mix (#V5072, Promega) according to the manufacturer instructions. After digest the samples were finally acidified to pH < 2 by adding trifluoroacetic acid. Desalting was done on C18 cartridges (SPE Columns C18/17%; #TR-F034000, Finisterre) according the manual instructions and concentrated to dryness in a speed vac. Peptides were analyzed on an EASY-nLC1000 system (Thermo Fischer Scientific) connected to a LTQ Orbitrap XL mass spectrometer (Thermo Fischer Scientific) as detailed below. Buffer A consisted of 3% acetonitrile, 0.1% formic acid and Buffer B of 63% acetonitrile, 0.1% formic acid. Dried peptide samples were resuspended in 50 μL Buffer A and 2 μL were separated in a 90 min gradient using an Acclaim^®^ PepMap RSLC analytical column (C18, 2 μm, 100 Å, 75 μm i.d. × 150 mm, #164534, Thermo Fischer Scientific). The gradient started from 3% to 15% acetonitrile in 30 min, to 30% acetonitrile over 60 min followed by a 10 min washout with 60% acetonitrile. The LTQ Orbitrap operated with a data dependent top 6 method as follows: MS full scans were performed in FTMS with resolution set to 60000, from 300.0 to 1600.0 m/z, a maximum fill time of 250 msec and an AGC target value of 1E6 ions. A maximum of 6 data dependent MS2 scans were performed in the ion trap set to an AGC target of 1E4 ions with a maximal injection time of 100 msec. Precursor ion fragmentation was achieved by collision induced fragmentation with a minimal signal of 250, isolation width of 2 m/z, normalized collision energy of 35, activation Q 0.25 and activation time 30. Charge states of 1 were rejected. Raw data were analyzed with MaxQuant version 1.5.6.0^17^ and its build-in search engine Andromeda^18^ using the default settings. The Arabidopsis protein database was downloaded from from Uniprot (http://www.uniprot.266org/proteomes/UP000006548) containing 31423 proteins, last modified May 14, 2016. The MaxQuant output is shown in Supplemental Table 5. To identify fraction specific proteins we required proteins to be present in the two replicates of one fraction, but to be absent in the respective other fraction.

### Western blot analysis

Proteins in fractions of one of the SEC experiments were analyzed by SDS-PAGE (10% Acrylamide). For this samples were loaded based on equal volume (15 μL + 3 μL 5x loading buffer). Proteins were blotted on a membrane and incubated with the primary antibody against Glutamine Synthetase (Anti-GLN1 GLN2 antibody Antisera (AS08295), 1:1000) in 5% Milk-TBST for 1 h at room temperature. The secondary antibody (goat anti-rabbit IgG –HPR, Antisera (AS09602), 1:20 000) was incubated for another hour at room temperature in 5% Milk-TBST. HRP signal was detected using SuperSignal™ West Pico Chemiluminescent Substrate (Thermo Fisher) as describe in the manual and exposed for 5 min.

## Additional Information

**How to cite this article**: Veyel, D. *et al*. System-wide detection of protein-small molecule complexes suggests extensive metabolite regulation in plants. *Sci. Rep.* **7**, 42387; doi: 10.1038/srep42387 (2017).

**Publisher's note:** Springer Nature remains neutral with regard to jurisdictional claims in published maps and institutional affiliations.

## Supplementary Material

Supplementary Material

Supplementary Table S1-S5
