# Supplementary material for: System-wide detection of protein-small molecule complexes suggests extensive metabolite regulation in plants
Source: Sci Rep. 2017 Feb 13;7:42387. doi: 10.1038/srep42387 (PMC5304321; doi:10.1038/srep42387)

System-wide detection of protein-small molecule complexes suggests extensive metabolite signaling in plants

Daniel Veyel , Sylwia Kierszniowska, Monika Chodasiewicz, Ewelina Sokolowsk, Aenne Michaelis, Marcin Luzarowski, Jagoda Szlachetko, Lothar Willmitzer, Aleksandra Skirycz

Supplementary information

Supplementary Table S1. Metabolite data of the size filtration experiments given as mean and standard deviation of n=5 technical replicates (same biological material was used in five independent filtration events). Relative intensity was normalized in all samples to the amount of starting material. Abbreviations RT – retention time in minutes.

Supplementary Table S2. Metabolite data of the size exclusion experiments given as mean and standard deviation of n=4 independent experiments. The raw data was normalized to its relative protein content in each fraction of different replicates.

Supplementary Table S3. Metabolite data of size filtration experiment testing the effect of heat denaturation.

Supplementary Table S4. Metabolite data of size filtration experiment testing the effect of NaCl concentrations on protein-bound small molecules.

Supplementary Table S5. Protein groups output table of LC-MS/MS proteomics analyses of SEC fractions B05 and C01 by MaxQuant.

Supplementary Figure S1. Heat denaturation has no major effect on the identity of small molecules found in the protein containing fraction. (a) Heatmap of metabolites found in the eluate sample of a size filtration experiment (referred to as protein bound) obtained with (Temp) or without heat denaturation (No temp) followed by drying and MTBE-Methanol-Water extraction. Out of 51 metabolites 39 were found in both treatments. (Mean, n=2). (b) Correlation of data from samples shown in (a) indicating minor effects of temperature treatment.

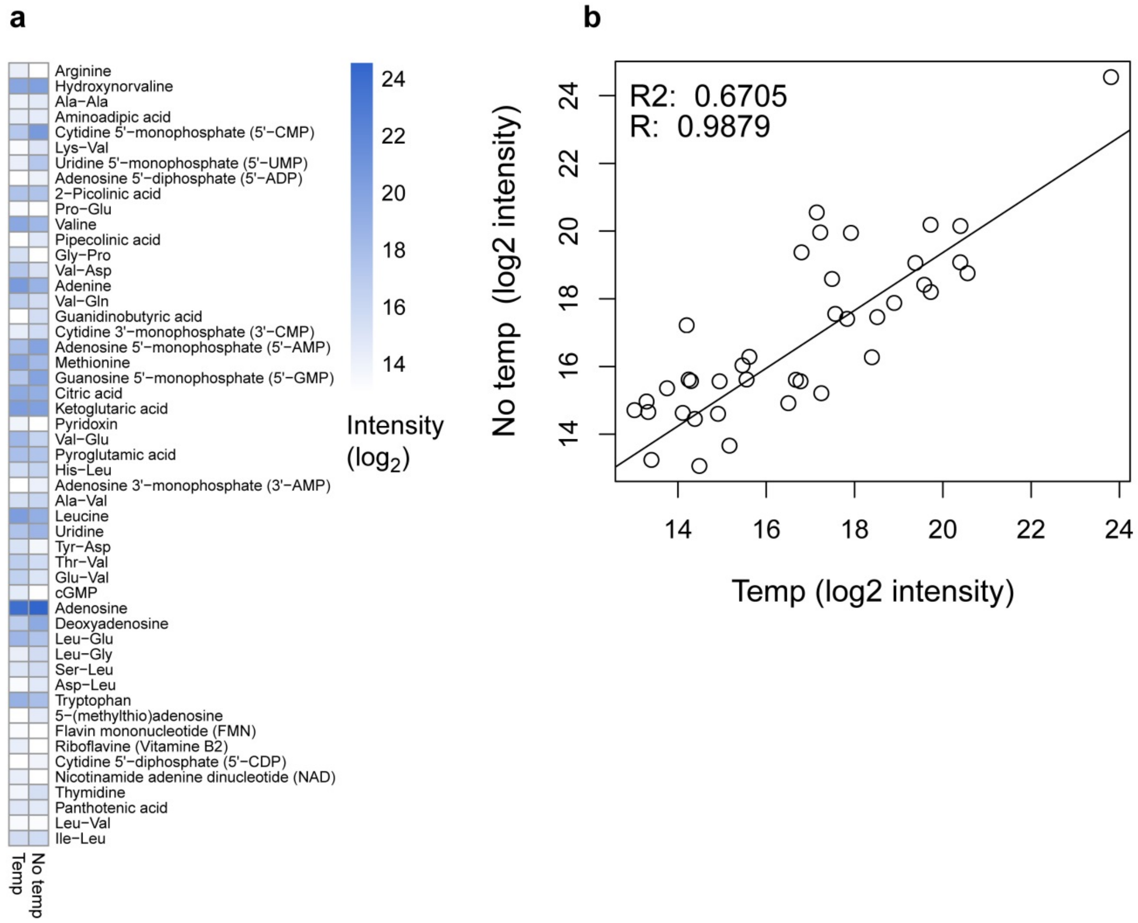

2

Supplementary Figure S2. Salt concentration in extraction buffer affects protein-small molecule interactions. (a) Heatmap presentation of metabolites found in the eluate sample (referred to as protein bound) using different salt concentrations in the lysis and wash buffers. (Mean, n=2). (b) Box plot showing the intensity distribution in the different samples indicating less small molecules remain protein bound at very high salt conditions.

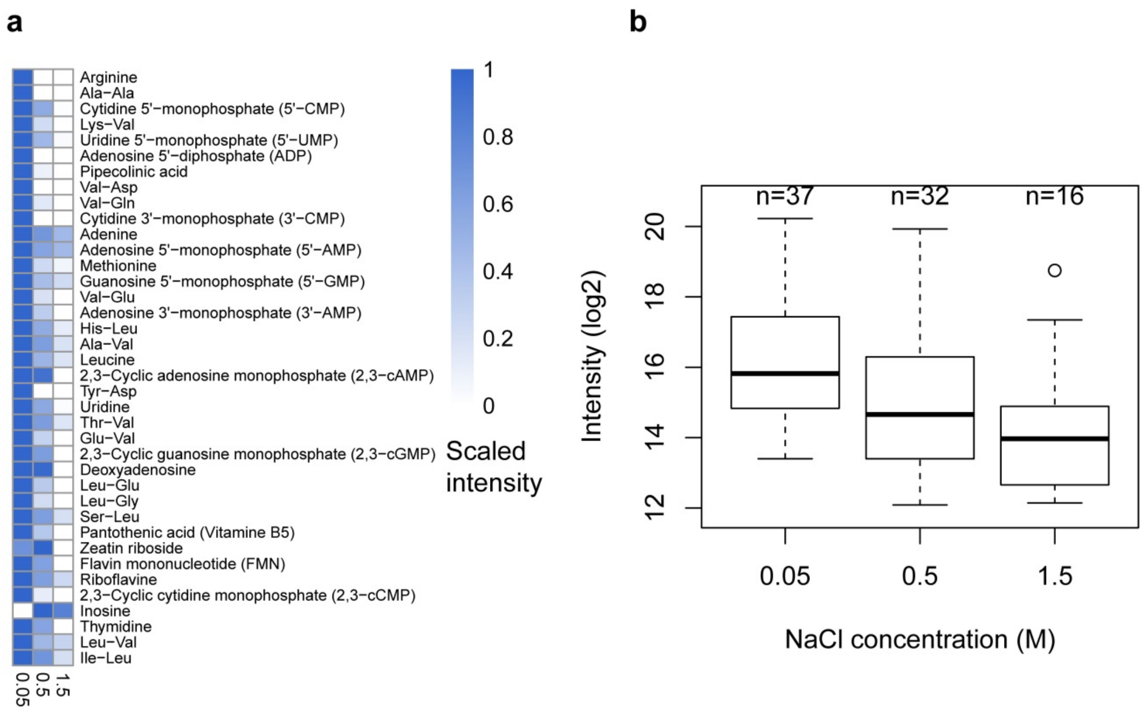

Supplementary Figure S3. Representative SDS-PAGE analysis of SEC fractions corresponding to MWs greater 10 kDa. 20 µL of each fraction were supplemented with 5x sample buffer and 17 µL were analyzed on a 10% polyacrylamide gel and Coomassie stained. The input was diluted tenfold and used as the SEC samples. Marker: SeeBlue® Plus2 Pre-stained (Thermo Fisher Scientific).

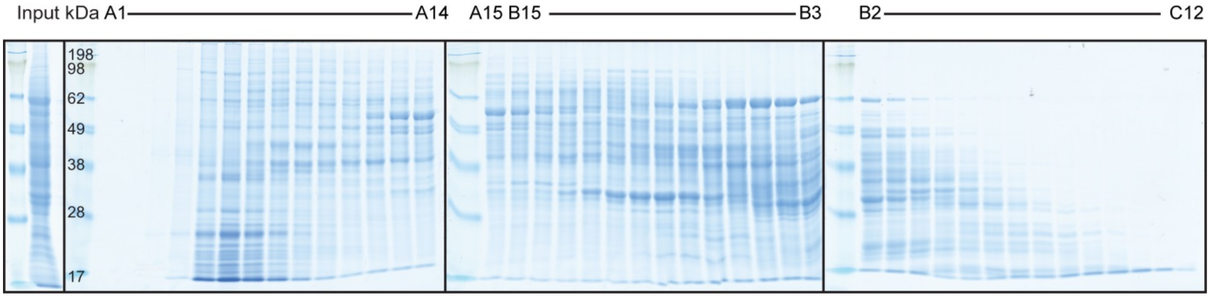

Supplement: Supplementary Material [file srep42387-s1.pdf]
